# Ion currents, action potentials, and noradrenergic responses in rat pulmonary vein and left atrial cardiomyocytes

**DOI:** 10.14814/phy2.14432

**Published:** 2020-05-13

**Authors:** Richard C. Bond, Stéphanie C. Choisy, Simon M. Bryant, Jules C. Hancox, Andrew F. James

**Affiliations:** ^1^ Cardiovascular Research Laboratories School of Physiology Biomedical Sciences Building University of Bristol Bristol UK

**Keywords:** action potential, delayed afterdepolarization, early afterdepolarization, inward‐rectifier K^+^ current (*I*_K1_), L‐type Ca^2+^ current (*I*_CaL_), noradrenaline, pulmonary vein sleeves, steady‐state K^+^ current (*I*_Kss_), triggered activity

## Abstract

The electrophysiological properties of pulmonary vein (PV)‐cardiomyocytes, and their responses to the sympathetic neurotransmitter, noradrenaline (NA), are thought to differ from those of the left atrium (LA) and contribute to atrial ectopy. The aim of this study was to examine rat PV cardiomyocyte electrophysiology and responses to NA in comparison with LA cells. LA and PV cardiomyocytes were isolated from adult male Wistar rat hearts, and membrane potentials and ion currents recorded at 36°C using whole‐cell patch‐clamp techniques. PV and LA cardiomyocytes did not differ in size. In control, there were no differences between the two cell‐types in zero‐current potential or action potential duration (APD) at 1 Hz, although the incidence of early afterdepolarizations (EADs) was greater in PV than LA cardiomyocytes. The L‐type Ca^2+^ current (*I*_CaL_) was ~×1.5 smaller (*p* = .0029, Student's *t* test) and the steady‐state K^+^ current (*I*_Kss_) was ~×1.4 larger (*p* = .0028, Student's *t* test) in PV than in LA cardiomyocytes. PV cardiomyocyte inward‐rectifier current (*I*_K1_) was slightly smaller than LA cardiomyocyte *I*_K1_. In LA cardiomyocytes, NA significantly prolonged APD_30_. In PV cells, APD_30_ responses to 1 μM NA were heterogeneous: while the mean percentage change in APD_30_ was not different from 0 (16.5 ± 9.7%, *n cells/N animals* = 12/10, *p* = .1177, one‐sample *t* test), three cells showed shortening (‐18.8 ± 6.0%) whereas nine showed prolongation (28.3 ± 10.1%, *p* = .008, Student's *t* test). NA had no effect on *I*_K1_ in either cell‐type but inhibited PV *I*_Kss_ by 41.9 ± 4.1% (*n/N* = 23/11 *p* < .0001), similar to LA cells. NA increased *I*_CaL_ in most PV cardiomyocytes (median × 2.2‐increase, *p* < .0001, *n/N* = 32/14, Wilcoxon‐signed‐rank test), although in 7/32 PV cells *I*_CaL_ was decreased following NA. PV cardiomyocytes differ from LA cells and respond heterogeneously to NA.

## Graphical abstract

Effects of noradrenaline on action potential (AP) repolarisation in PV cardiomyocytes. A Representative AP recording from a PV cardiomyocyte in control solution and in the presence of 1 μM noradrenaline (NA). B Representative AP recording from another PV cardiomyocyte in control solution and in the presence of 1 μM NA showing lack of AP prolongation. C Before‐and‐after plot of APD_30_ in PV cardiomyocytes in control solution and after superfusion with 1 μM NA. D Noradrenaline‐induced fold‐changes in APD_30_ in LA and PV cardiomyocytes.

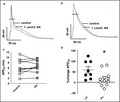

## 1 INTRODUCTION

Atrial fibrillation (AF) is a rapid and irregular activation of the atria that can lead to serious clinical consequences, including reduced left ventricular function, irregular ventricular rhythm, heart failure, and stroke, and is associated with a significantly elevated risk of death (Camm et al., 2010). AF is the most common cardiac arrhythmia, the incidence increasing with age, being present in between 1% and 2% of the general population, rising to 3%–5% in those over 65 years of age, and reaching 15% in octogenarians (Camm et al., 2010). AF is generally considered to be a progressive condition; patients initially presenting with paroxysmal AF progress to longer, non‐self‐terminating bouts (Camm et al., 2010). It is widely accepted that the progressive nature of AF is due to atrial remodeling caused by AF itself establishing a substrate for reentry (Schotten, Verheule, Kirchhof, & Goette, 2011; Wakili, Voigt, Kääb, Dobrev, & Nattel, 2011). Early intervention to restore and maintain normal sinus rhythm (SR) is therefore desirable.

The myocardial sleeves of the pulmonary veins (PVs) are a major source of ectopic activity triggering AF (Jaïs et al., 2002). Isolation of the PVs from the LA by catheter ablation has been shown to be effective in restoring and maintaining SR in AF patients (Oral et al., 2006; Pappone et al., 2006). It has been suggested that the electrophysiological properties of cardiomyocytes from the PV sleeves render them susceptible to arrhythmogenesis (Chen & Chen, 2006; Nattel, 2003). Nodal‐like cells have been identified in microscopy studies of rat, canine, and human PVs (Chou et al., 2005; Masani, 1986; Perez‐Lugones et al., 2003). Spontaneous action potentials preceded by prominent pacemaker‐type diastolic depolarizations have been recorded from intact PV preparations from laboratory animals in vitro (Chen, Chen, Chang, & Lin, 2000; Chen, Chen, Chen, Chang, et al., 2002; Chen et al., 2001; Chen, Chen, Chen, Yeh, et al., 2002; Cheung, 1981b; Chou et al., 2005; Miyauchi et al., 2005; Patterson, Po, Scherlag, & Lazzara, 2005), although some groups have reported being unable to detect pacemaker‐type activity under resting conditions (Cheung, 1981a; Ehrlich et al., 2003; Hocini et al., 2002). It has been suggested that the lower background K^+^ conductance, more depolarized resting membrane potential and shorter action potential duration (APD) of PV cardiomyocytes compared to LA cardiomyocytes, together with differences in fiber orientation in the vicinity of the PVs, may render this region more susceptible to abnormal automaticity, triggered activity and reentry (Chen & Chen, 2006; Ehrlich et al., 2003; Nattel, 2003).

Autonomic innervation is thought to play an important role in initiating paroxysms of AF, sympathetic stimulation being particularly significant in patients with heart disease (Arora, 2012; Chen, Chen, Fishbein, Lin, & Nattel, 2014; Coumel, 1996). The sympathetic neurotransmitter, noradrenaline (NA), has been shown to induce automatic activity in in vitro preparations of the PV, but not LA, from rats (Doisne, Maupoil, Cosnay, & Findlay, 2009; Maupoil, Bronquard, Freslon, Cosnay, & Findlay, 2007). The effect of NA was associated with a slowly developing depolarization of the resting membrane preceded by a transient hyperpolarization (Doisne et al., 2009). Noradrenaline has previously been shown to cause APD prolongation through potentiation of the L‐type Ca^2+^ current (*I*_CaL_) and inhibition of a TREK‐1 channel‐like steady‐state outward current (*I*_Kss_) in rat isolated LA cardiomyocytes (Bond, Choisy, Bryant, Hancox, & James, 2014). The objective of this study was to characterize the electrophysiological properties of isolated PV cardiomyocytes and their responses to NA in comparison with LA cardiomyocytes from rat hearts.

## 2 MATERIALS AND METHODS

### 2.1 Animals and cardiomyocyte isolation

Left atrial and PV sleeve cardiomyocytes were isolated from adult male Wistar rats (200–320 g). All procedures were conducted in accordance with UK legislation, were approved by the *University of Bristol Animal Welfare and Ethics Review Board* and were conducted in accordance with the ARRIVE Guidelines. LA cardiomyocytes were isolated following retrograde perfusion of the aorta with a collagenase‐containing Tyrode's solution (Type 1, Worthington, 205–351 U/mg) using a modified Langendorff apparatus, as follows (Bond et al., 2014, 2017). Following general anesthesia (intraperitoneal injection of 60–100 mg/kg sodium pentobarbital and 625 international units (IU) of heparin), the heart was excised rapidly and placed in a dunk beaker containing solution A (see below) plus 750 μM CaCl_2_ and 10 IU of heparin (pH 7.4, room temperature, ~22°C). The heart was then mounted on a Langendorff apparatus and retrogradely perfused via the aorta at a flow rate of 8 ml·min^‐1^·g^‐1^ of heart tissue. Solution A contained (in mM): 130 NaCl, 5.4 KCl, 4.2 4‐(2‐hydroxyethyl)‐1‐piperazineethanesulfonic acid (HEPES), 10 glucose, 20 taurine, 10 creatine, 1.4 MgCl_2_, and 0.4 NaH_2_PO_4_; pH 7.61 with NaOH to give a pH of 7.4 at 37°C. This solution formed the basis for three separate isolation solutions, each of which was loaded to the reservoir vessels of the Langendorff apparatus, warmed to 37°C, and oxygenated. The heart was first perfused for 4 min with solution 1 consisting of solution A plus 750 μM CaCl_2_. During this time, the heart typically cleared of blood and beat in a regular manner. The heart was then perfused for 4 min with solution 2 comprising the nominally Ca^2+^‐free solution A plus the calcium chelator, ethylene glycol tetraacetic acid (EGTA) (100 μM). During this time the heart stopped beating and the atria started to swell. Finally, the heart was perfused with solution 3 consisting of solution A plus 50 – 240 μM CaCl_2_ and 1 mg/ml collagenase (Type 1, Worthington, 205–351 U/mg). Typically, the atria continued to swell and then collapsed. After 12 min of perfusion with the enzyme containing solution, the LA was removed into a Kraftbrühe (KB) solution consisting of (in mM): 90 l‐glutamic acid, 30 KCl, 10 HEPES, 1 EGTA, 5 Na pyruvate, 20 taurine, 20 glucose, 5 MgCl_2_, 5 succinic acid, 5 creatine, 2 Na_2_ATP and 5 β‐OH butyric acid; pH 7.4 with KOH (Isenberg & Klockner, 1982). The LA was then finely chopped and triturated with a fire‐polished glass Pasteur pipette. Dissociated cells were stored in KB at 4°C and used within 10 hr of isolation.

PV cardiomyocytes were isolated using a modification of this method using the same solutions: the lungs were excised together with the heart *en bloc* and retained during the Langendorff perfusion of the heart. After perfusion of the heart with the collagenase solution for 9 min, the heart and lungs were removed into a Sylgard‐lined Petri dish and the tissue pinned. The PV sleeves were identified using a binocular dissecting microscope and carefully dissected from the posterior LA to the lung hilum. The proximal portion of the sample (~2 mm) was discarded to avoid contamination with LA cardiomyocytes. The PV was cut in half and placed in a second enzyme solution containing 1 mg/ml collagenase (Type 1, Worthington, 205–210 U/mg), 0.5 mg/ml papain (Sigma‐Aldrich), 0.7 mg/ml dl‐dithiothreitol (Sigma), 160 μl 10% BSA, and 50–240 μM CaCl_2_. This was then shaken in a water bath at 37°C for 14 min. The PVs were then removed, finely chopped, and triturated using a fire‐polished glass Pasteur pipette in KB solution (Isenberg & Klockner, 1982). Dissociated LA and PV cardiomyocytes were stored in KB at 4°C and used within 10 hr of isolation.

### 2.2 Measurement of cardiomyocyte length and width

**Figure 1:**
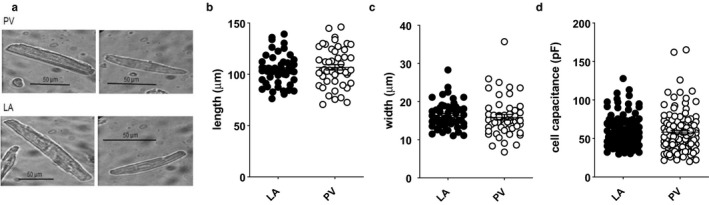
Sizes of left atrial (LA) and pulmonary vein (PV) cardiomyocytes. (a) Representative photomicrographs of PV (upper images) and LA (lower images) cardiomyocytes. Scale bars as indicated. (b) Cell lengths of LA (filled circles, *n/N* = 50/13) and PV (open circles, *n/N* = 50/17) cardiomyocytes. Horizontal lines indicate mean values with error bars showing standard error of the mean (*SEM*). (c) Cell widths of LA (filled circles, *n/N* = 50/13) and PV (open circles, *n/N* = 50/17) cardiomyocytes. Horizontal lines indicate mean values with error bars showing *SEM*. (d) Whole‐cell capacitances of LA (filled circles, *n/N* = 125/37) and PV (open circles, *n/N* = 125/53) cardiomyocytes. Horizontal lines indicate mean values with error bars showing *SEM*

Cardiomyocyte mean length and width were determined by measuring 50 cardiomyocytes (13 hearts for LA and 17 hearts for PV) from images taken on a digital camera (MyoCam S, IonOptix) attached to a Nikon Diaphot 400 inverting microscope (Nikon UK Ltd.). Representative images of LA and PV cardiomyocytes are shown in Figure 1a. Images were analyzed using ImageJ 1.48 software (*National Institutes of Health, USA*). The mean cardiomyocyte volume was calculated assuming that the cardiomyocyte was an elliptical cylinder with a depth to width ratio of 1:3 (Boyett, Frampton, & Kirby, 1991; Sorenson, Tepper, Sonnenblick, Robinson, & Capasso, 1985). The volume of an elliptical cylinder is given by

(1) $$\text{Vol}=\frac{\pi wdl}{4}$$

where *w* represents width, *d* is depth, and *l* is length.

#### 2.2.1 Whole‐cell recording

Isolated cardiomyocytes were placed in a chamber on the stage of an inverted microscope and superfused with external solution comprising (in mM) 140 NaCl, 4 KCl, 1 MgCl_2_, 2.5 CaCl_2_, 10 d‐glucose, and 5 HEPES, pH 7.4 at 36°C, as described previously (Levi, Brooksby, & Hancox, 1993). In some experiments, the inwardly rectifying K^+^ current (*I*_K1_) was isolated by the superfusion of the cells with a K^+^‐free external solution in which KCl had been replaced with equimolar CsCl. For whole‐cell voltage‐clamp recording, pipettes were filled with a solution comprising (in mM) 10 NaCl, 110 KCl, 0.4 MgCl_2_, 5 d‐glucose, 10 HEPES, 5 1,2‐bis(*o*‐aminophenoxy)ethane‐N,N,N',N'‐tetraacetic acid (BAPTA), 5 adenosine 5'‐triphosphate dipotassium salt dihydrate (K.ATP), and 0.5 guanosine 5′‐triphosphate tris salt (Tris.GTP), pH 7.3 (KOH). For K^+^‐free experiments, KCl in both internal and external solutions was replaced with equimolar CsCl. For current clamp recordings, a nominally Ca^2+^‐free K^+^‐based pipette solution was used according to the recipe above but without addition of BAPTA. Patch pipettes (borosilicate glass, A‐M systems, USA) were pulled (P‐97 Flaming‐Brown, Sutter Instruments, USA) to resistances of 1–2 MΩ and filled with the appropriate internal solution using a Microfil syringe (World Precision Instruments, USA).

Recording protocols were generated by, and currents and voltages recorded to a PC running Pulse software (versus 8.11, HEKA GmbH, Germany) using an EPC‐9 (HEKA GmbH, Germany), or an Axopatch 200B patch clamp amplifier (Molecular Devices Ltd., UK) coupled with a PCI‐16 interface (Instrutech Inc., USA). The junction current was zeroed on inserting the pipette into the external solution. The junction potential was calculated (Axon Instruments pClamp10, Molecular Devices, UK) to be ‐4.8 mV and no further correction was applied. Recordings were only made from cells with a seal resistance of ≥1 GΩ on formation of the cell‐attached configuration prior to whole‐cell access. Action potentials (APs) were recorded at 36°C after whole‐cell access had been obtained. LA cardiomyocytes were voltage clamped to the anticipated resting membrane potential (RMP) of ‐80 mV and PV cardiomyocytes at ‐75 mV before switching to current clamp mode on the amplifier. The membrane potential was recorded at zero current and, if different from the anticipated RMP (~‐80 mV for LA cardiomyocytes and ~‐75 mV for PV cardiomyocytes), current was injected to bring RMP close to the expected value for that cell type (Cha et al., 2005; Chen, Chen, Chen, Yeh, et al., 2002; Ehrlich et al., 2003; Ertl, Jahnel, Nawrath, Carmeliet, & Vereecke, 1991; Komukai, Brette, & Orchard, 2002; Okamoto, Takano, Ohba, & Ono, 2012; Tavi, Han, & Weckstrom, 1998). APs were elicited at a frequency of 1 Hz by depolarizing current pulses, as has been described previously (Cha et al., 2005; Chen, Chen, Chen, Yeh, et al., 2002; Ehrlich et al., 2003). The incidence of delayed and early afterdepolarizations (respectively, DADs and EADs) and triggered activity (TA) under control conditions was recorded over a period of 1 min. A DAD was defined as a deviation of the baseline by ≥ 2 mV occurring after the membrane potential had returned to the baseline following a stimulated action potential. An EAD was defined as a positive deviation from the normal course of repolarization. Triggered activity was defined as an action potential that was produced by an afterdepolarization. The effect of NA on the frequency of DADs and EADs was examined by recording under control conditions, followed by recording for >90 s during superfusion with 1 µM NA.

In voltage‐clamp experiments, capacitance and series resistance were routinely compensated, with series resistance compensation of ~70%. Capacitance was recorded as an index of cell surface area. Voltage‐gated inward and outward currents were investigated using a square wave pulse protocol: Cardiomyocyte membrane potential was clamped to a holding potential of ‐80 mV. Following a step to ‐40 mV (50 ms) to inactivate *I*_Na_, 500 ms depolarizing pulses were applied to potentials from ‐40 mV to +50 mV increasing in 10 mV increments every 5 s (inset, Figure 4a). The voltage‐dependent inward current was calculated as a difference current by subtracting the outward current at the end of the pulse from the peak inward current obtained. The “steady‐state” outward current was taken as the current at the end of the pulse. A ramp protocol was also used to investigate the steady‐state outward current and inwardly rectifying current (inset, Figure 6a). Cardiomyocytes were held at a holding potential of ‐80 mV. Every 3 s a step to +20 mV for 100 ms was followed by a ramp to ‐120 mV over 500 ms. Current densities were calculated by normalization to capacitance as an index of cell surface area.

Noradrenaline was used at 1 μM, as this has been shown previously to produce near maximal potentiation of L‐type Ca^2+^ current (*I*_CaL_) in LA cardiomyocytes and is likely to be representative of the concentrations of the neurotransmitter at the cardiac sympathetic neuroeffector junction (Bond et al., 2014; Goldstein, McCarty, Polinsky, & Kopin, 1983).

### 2.3 Data analysis and statistics

Data were stored on the hard drive of a computer and analyzed offline using Igor Pro software (versus 3.16B, WaveMetrics Inc, USA). The mean current density–voltage relations for the voltage‐gated inward current (*I*_pk‐in_) were fitted with a modified Boltzmann equation, as follows:

(2) $$I_{pk-in}\left(V_{m}\right)=\frac{G_{\max}.\left(V_{m}-V_{\mathrm{rev}}\right)}{\left(1+e^{\left(\frac{\left(V_{\mathrm{half}}-V_{m}\right)}{k}\right)}\right)}$$

where *V_m_* represents the membrane potential, *G*_max_ is the maximum conductance of the membrane, *V_rev_* is the potential at which the current reverses direction, *V_half_* represents the voltage of half‐maximal activation, and *k* is a slope factor.

Figures were created and statistical analysis performed using Prism software (version 7.04, GraphPad Software Inc.). Data were subject to D’Agostino–Pearson normality test. Where appropriate, data are presented as the mean ± S.E.M and compared statistically using Student's *t* test, Mann–Whitney test, or one‐way or two‐way analysis of variance (ANOVA), with repeated measures (RM). Bonferroni post hoc tests were used, with correction for multiple comparisons where appropriate.

For data that were normally distributed, such as the NA‐induced prolongation of action potential duration at 30% repolarization (APD_30_) shown in Figure 7, the coefficient of variation (*CoV_s_*) was calculated as:

(3) $$CoV_{s}=\left(s\;/\bar{x}\right)\times 100$$

where *s* represents the standard deviation of the sample and $\bar{x}$ is the sample mean. The *CoV_s_* of two samples were compared by calculation for each sample the ratio of the absolute deviations from the mean of each observation, normalized to the mean of that sample, as follows:

(4) $${\text{Ratio}}_{i}=\frac{\left|x_{i}-\bar{x}\right|}{\bar{x}},$$

where *x_i_* represents the ith observation of a sample and $\bar{x}$ is the sample mean. A Levene's *F* test for homogeneity of variance was then performed on the Ratio_i_ values to compare the two samples (Schultz, 1985).

In contrast, for data that were not normally distributed, such as the NA‐induced fold‐change in *I*_CaL_ shown in Figure 9, the interquartile range normalized to the median was calculated as an interquartile coefficient of variation (*CoV_iq_*), as follows:

(5) $$CoV_{\mathrm{iq}}=\left(\frac{Q_{3}-Q_{1}}{Q_{2}}\right)\times 100,$$

where *Q*_1_ and *Q_3_* are the first and third quartiles, and *Q_2_* is the median. In a manner analogous to the comparison of *CoV_s_*, the *CoV_iq_* of the two samples were then compared by calculation for each sample the ratio of the absolute deviations from the median of each observation, normalized to the median of that sample, as follows:

(6) $${\text{Ratio}}_{i}=\frac{\left|x_{i}-Q_{2}\right|}{Q_{2}},$$

where *x_i_* represents the ith observation of a sample and *Q_2_* is the sample median. A Brown–Forsythe test was then performed as an *F*‐test on these values to compare the two samples (Brown & Forsythe, 1974).

Sample sizes are reported as “*n”* numbers of cells from “*N”* numbers of animals (i.e., *n/N*). Confidence tests were applied using *n* numbers of cells as the sample size. Contingency data regarding the incidence of EADs, DADs, and TA were analyzed using Fisher's exact test. A *p* value of < .05 was considered significant.

## 3 RESULTS

### 3.1 Morphological appearance of PV and LA cardiomyocytes

Cardiomyocytes from rat PV and LA preparations had a similar appearance, with clear striations visible, and both cell‐types were rod‐shaped with square or tapered ends (Figure 1a). There were no significant differences between PV and LA cardiomyocytes in cell length or cell width (Figure 1b & Figure 1c). It follows that there was no difference between the two cell types in the mean cell volume, calculated using *equation* *1* (PV cardiomyocytes 7,978 ± 848 μm^3^, *n/N* = 50/13 and LA cardiomyocytes 7,799 ± 569 μm^3^, *n/N* = 50/17; *p* = .4645 Mann–Whitney test). There was no difference in the whole‐cell capacitance, a measure of cell surface area, obtained from voltage‐clamp recordings (Figure 1d).

### 3.2 Action potentials in PV and LA cardiomyocytes

**Figure 2:**
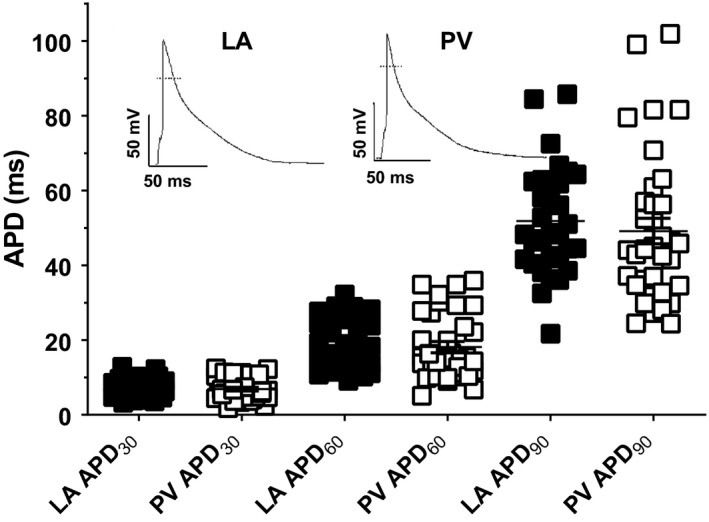
Action potential durations (APD) in LA and PV cardiomyocytes. APD are shown at 30%, 60% and 90% repolarization of the AP amplitude in LA (filled squares, *n/N* = 32/9) and PV (open squares, *n/N* = 35/14) cardiomyocytes. Following current injection (see text for details), the mean RMP were LA cardiomyocytes, −81 ± 0.1 mV (*n/N* = 24/7) and PV cardiomyocytes −74 ± 0.1 mV(*n/N* = 22/9). Inset shows representative recordings from LA and PV cardiomyocytes. Stimulus frequency was 1 Hz

**Figure 3:**
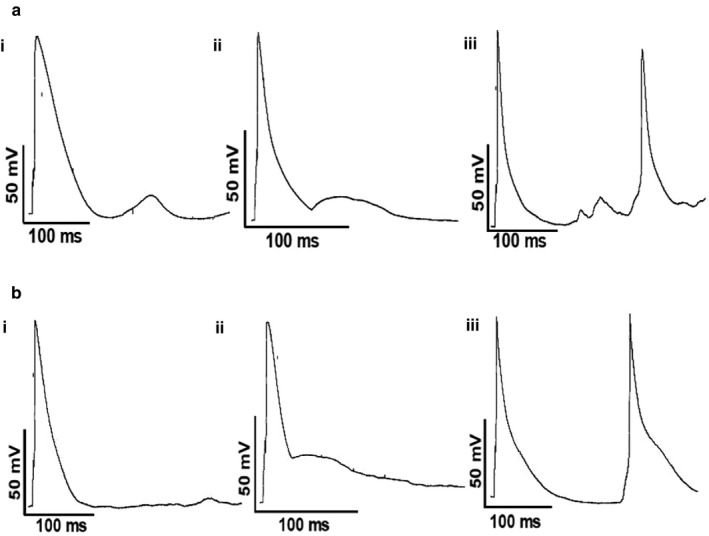
Proarrhythmic events in LA and PV cardiomyocytes. (a) Representative recordings of (i) delayed afterdepolarization, (ii) early afterdepolarization and (iii) triggered activity recorded from LA cardiomyocytes. (b) Representative recordings of (i) delayed afterdepolarization, (ii) early afterdepolarization and (iii) triggered activity recorded from PV cardiomyocytes. The stimulation frequency was 1 Hz

Prior to whole‐cell recording, while being superfused with control Tyrode's solution rat cardiomyocytes of either cell type were quiescent, showing no evidence of regular spontaneous contractile activity. There was no difference in zero‐current potential between LA cardiomyocytes (LA ‐56.3 ± 3.2 mV, *n/N* = 24/7; PV cardiomyocytes ‐53.8 ± 3.3 mV, *n/N* = 22/9). At the zero‐current potential, no spontaneous electrical activity was observed and it was not possible to stimulate the cells to produce action potentials. It was assumed that the normal resting membrane potential (RMP) of LA cardiomyocytes was close to ‐80 mV (Ertl et al., 1991; Komukai et al., 2002; Tavi et al., 1998) and so current was injected to LA cardiomyocytes (44 ± 7.1 pA, *n/N* = 24/7) to achieve a mean RMP of ‐81 ± 0.1 mV. PV cardiomyocytes have been reported to have a RMP that is 5–10 mV less negative than that of LA cardiomyocytes (Cha et al., 2005; Chen, Chen, Chen, Yeh, et al., 2002; Ehrlich et al., 2003; Okamoto et al., 2012) and so current was injected (66 ± 10.1 pA, *n/N* = 22/9) to PV cardiomyocytes to achieve a mean RMP of ‐74 ± 0.1 mV (*n* = 22).The difference between LA and PV cardiomyocytes in injected current was not statistically significant (*p* = .0778, Student's unpaired *t* test). In LA and PV cardiomyocytes, the action potential profiles were generally similar and triangular in shape (inset, Figure 2). The mean action potential duration (APD) at 30% (APD_30_), 60% (APD_60_) and 90% (APD_90_) of repolarization were not significantly different between LA and PV cardiomyocytes (Figure 2). During recording of APs, delayed and early afterdepolarizations (respectively, DADs and EADs) and triggered activity (TA) were recorded from both LA and PV cardiomyocytes (Figure 3). Under control conditions, the incidence of proarrhythmic events in LA cardiomyocytes was low in comparison to PV cardiomyocytes (Table 1). Notably, the incidence of EADs was significantly greater in PV cardiomyocytes than in LA cardiomyocytes (Table 1).

**Table 1:** Incidence of proarrhythmic events in isolated LA and PV cardiomyocytes. Numbers in parentheses show the percentage of the total number of cells showing proarrhythmic event

	Total No. of cells/animals	No. of cells (%) with DADs	No. of cells (%) with EADs	No. of cells (%) with TA
LA—control	25/10	4 (16)	3 (12)	1 (4)
PV—control	15/10	7 (46)	8** (53)	1 (7)

** *p* < .01, Fisher's exact test compared with incidence in LA cardiomyocytes

### 3.3 Differences in ion currents between LA and PV cardiomyocytes

**Figure 4:**
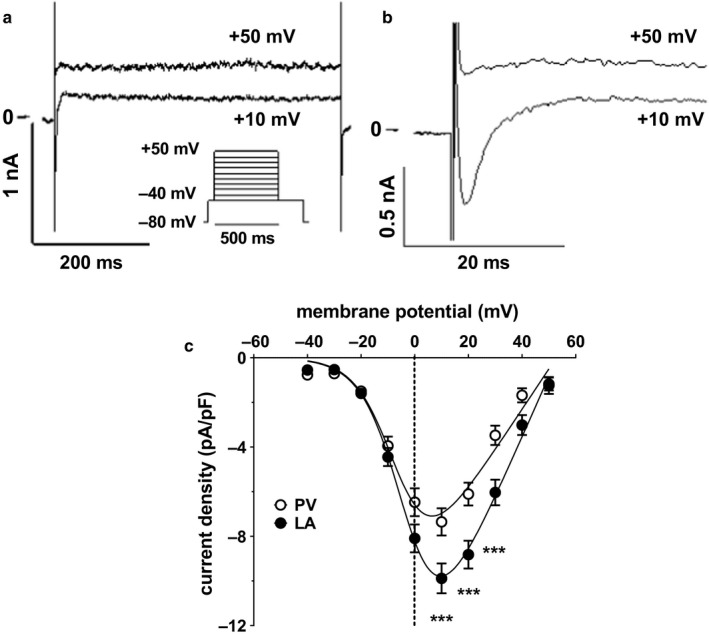
Differences in peak inward current density between LA and PV cardiomyocytes. (a) Representative currents from an LA cardiomyocyte obtained from square wave pulse protocol (shown inset) at +10 mV and +50 mV demonstrating an inward current that rapidly reached a peak and subsequently inactivated to a steady‐state outward current level by the end of the pulse and a steady‐state outward current that showed little inactivation at the end of the pulse. (b) Data shown in (a) on expanded time and current scales showing the currents in more detail at +10 mV and +50 mV. (c) Mean *I*_CaL_ density‐voltage relations for PV cardiomyocytes (open circles, *n/N* = 38/16) and LA cardiomyocytes (filled circles, *n/N* = 39/12). Solid lines represent fits to *equation* *2*.***p ≤ .001; repeated measures two‐way ANOVA with Bonferroni post hoc test

**Figure 5:**
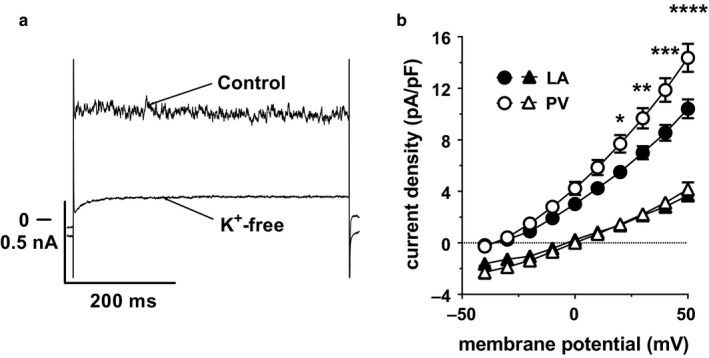
Differences in steady‐state outward current density between LA and PV cardiomyocytes. (a) Representative current traces from a PV cardiomyocyte in the presence (control) and absence (K^+^‐free) of internal and external K^+^ obtained using the square wave pulse protocol at +50 mV (protocol as in Figure 3a). (b) Mean steady‐state outward current density‐voltage relations for PV cardiomyocytes in control (open circles, *n/N* = 35/15) and K^+^‐free (open triangles, *n/N* = 7/3) conditions and LA cardiomyocytes in control (filled circles, *n/N* = 41/13) and K^+^‐free (filled triangles, *n/N* = 10/3) conditions.*, *p* ≤ .05; **, *p* ≤ .01; ***, *p* ≤ .001; ****, *p* ≤ .0001; PV versus LA cardiomyocytes in control conditions, repeated measures two‐way ANOVA with Bonferroni post hoc test. Under control conditions, data were different by membrane potential (*p* < .0001) and cell type (*p* = .0079), with a significant interaction (*p* < .0001). For the sake of clarity, the results of statistical comparison of current densities recorded from either cell type between control and K^+^‐free recording conditions were not shown in the figure. For PV cardiomyocytes, the data were significantly different by the presence/absence of K^+^ (*p* = .0001), by voltage (*p* < .0001) and there was a significant interaction (*p* < .0001). In Bonferroni post hoc test, there were significant differences between the mean data from +10 mV to +50 mV (*p* from .0069 to <.0001). For LA cardiomyocytes, the data were significantly different by the presence/absence of K^+^ (*p* < .0001), by voltage (*p* < .0001) and there was a significant interaction (*p* < .0001). In Bonferroni post hoc test, there were significant differences between the mean data from 0 mV to +50 mV (*p* from .0099 to <.0001)

As reported previously in rat LA cardiomyocytes using recording conditions identical to those used in the present study (Bond et al., 2014), two distinct current components were activated during square‐shaped depolarizing pulses (500 ms) from a holding potential of ‐80 mV to potentials of ‐30 mV and positive: (a) an inward current that rapidly reached a peak and subsequently inactivated to a steady‐state outward current level by the end of the pulse and (b) a steady‐state outward current that showed little inactivatiossn at the end of the pulse (Figure 4a). The inward current corresponds to the L‐type Ca^2+^ current (*I*_CaL_) while the steady‐state outward current (*I*_Kss_) is carried predominantly by K^+^ (Bond et al., 2014). In both PV and LA cardiomyocytes, the maximal *I*_CaL_ density was at +10 mV (Figure 4b). *I*_CaL_ density was significantly smaller in PV cardiomyocytes (*n/N* = 38/16) compared to LA cardiomyocytes (*n/N* = 39/12) at voltages of +10 mV to +30 mV (Figure 4c). The maximal conductance density (*G_max_*), fitted using *equation* *2*, was approximately ×1.5 smaller in PV than in LA cardiomyocytes (Table 2). However, there were no significant differences in the biophysical properties of *I*_CaL_ (*i.e., V_rev_*, *V_half_*, *k*) between the two cell types (Table 2). In the presence of K^+^, the steady‐state outward current density was greater in PV than in LA cardiomyocytes, this effect being most marked at positive voltages (Figure 5). In both cell types, removal of K^+^ was associated with a marked reduction in current densities at all voltages and a shift in the apparent reversal potential to the right (Figure 5b). Notably, in the absence of K^+^, there was no significant difference in current densities between cell types (Figure 5b; two‐way RM ANOVA, *p* = .6440), indicating a difference in *I*_Kss_ density between PV and LA cardiomyocytes.

**Table 2:** Parameters fitted to the *I_CaL_* density‐voltage relation data shown in Figure 3c using equation 2

	PV cardiomyocytes	LA cardiomyocytes	*p*
*G_max_*(pS/pF)	181 ± 17.8	272 ± 23.1	.0029
*V_rev_*(mV)	53 ± 2.1	53 ± 1.6	.9007
*V_half_*(mV)	‐5.7 ± 1.6	‐2.1 ± 1.4	.0939
*k*(mV)	7.1 ± 0.9	7.4 ± 0.8	.8160

Values are fitted parameters ± standard error of fitting. The data were obtained from 38 PV and 39 LA cardiomyocytes from, respectively, 16 and 12 animals.

**Figure 6:**
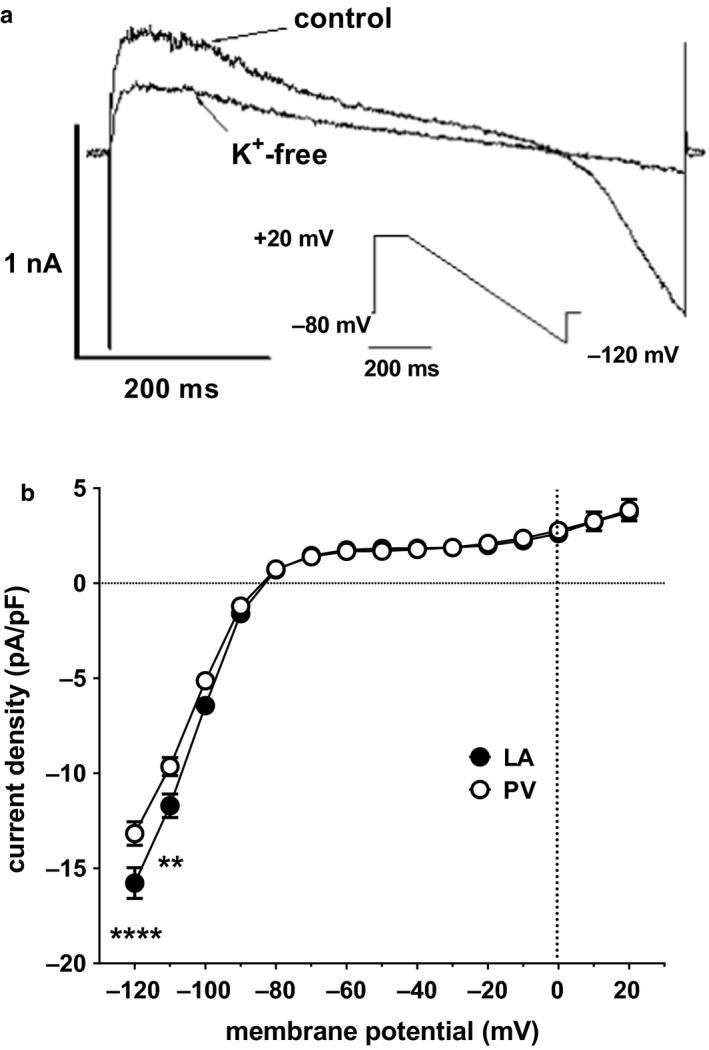
Background K^+^ currents in PV and LA cardiomyocytes. (a) Representative currents from a PV cardiomyocyte obtained from the ramp protocol (shown inset) in control conditions ([K^+^]_e_ = 4 mM) and external K^+^‐free conditions ([K^+^]_e_ = 0). The currents were recorded from the same cell. (b) Mean K_e_^+^‐sensitive background K^+^ current density‐voltage relations obtained after subtraction of ramp currents recorded in external K^+^‐free conditions from currents recorded in the presence of 4 mM [K^+^]_e_ in PV (open circles, *n/N* = 26/11) and LA cardiomyocytes (filled circles, *n/N* = 50/24). **p < .01; ****p < .0001; two‐way RM ANOVA with Bonferroni post hoc test

The ramp protocol allowed the simultaneous measurement of the steady‐state outward (*I*_Kss_) and inwardly rectifying K^+^ current (*I*_K1_) (Bond et al., 2014). Step depolarization to + 20 mV produced a large, fast, and poorly resolved inward current that rapidly inactivated to a noisy outward current at the end of the 100 ms step (Figure 6a). During the descending voltage ramp to ‐120 mV, currents showed inward rectification, which was particularly marked at negative potentials (Figure 6a). Both *I*_Kss_, predominant at +20 mV, and *I*_K1_, predominant at voltages negative to the expected reversal potential for K^+^‐selective currents (E_K_≈‐90 mV), were sensitive to removal of external K^+^ (K^+^_e_). The inwardly rectifying current was effectively abolished on removal of external K^+^, consistent with the contributions of inwardly rectifying K^+^ channels (K_ir_) to *I*_K1_ (Hibino et al., 2010; Yan et al., 2005). Note that while the strong inward rectification of *I*_K1_ recorded from LA and PV cardiomyocytes is consistent with a major contribution of K_ir_2.x in either cell type, in principle, constitutively active G‐protein‐gated K_ir_3.x channels and ATP‐sensitive K_ir_6.x/SURx (K_ATP_) channels may also have contributed to the K^+^_e_‐sensitive inwardly rectifying currents in the present study (Ehrlich et al., 2004; Hibino et al., 2010; Kim et al., 2012). In addition, a component of *I*_Kss_, evident at positive potentials using the ramp protocol, was also inhibited by removal of external K^+^. Thus, the mean K^+^_e_‐sensitive difference current in Figure 6b shows the contribution of both *I*_K1_ at potentials of ‐90 mV and negative and a K^+^_e_‐sensitive component of *I*_Kss_ at positive potentials. *I*_K1_ was smaller in PV than in LA cardiomyocytes, albeit reaching statistical significance outside of the usual physiological range at ‐110 mV and ‐120 mV (respectively, *p* < .01 and *p* < .0001) (Figure 6b). Notably, there was no significant difference between LA and PV cardiomyocytes in the K^+^_e_‐sensitive component of *I*_Kss_ at +20 mV. Interestingly, the sensitivity of *I*_Kss_ to removal of K^+^_e_ is consistent with the contribution of TREK‐1 to these currents, as suggested previously (Bond et al., 2014; Ma et al., 2011). However, further work is required to confirm the contribution of TREK‐1 to *I*_Kss_ and whether the differences in *I*_Kss_ density between rat LA and PV cardiomyocytes could be accounted for by differences in TREK‐1 protein expression at the sarcolemma.

### 3.4 Responses of PV cardiomyocytes to noradrenaline

**Figure 7:**
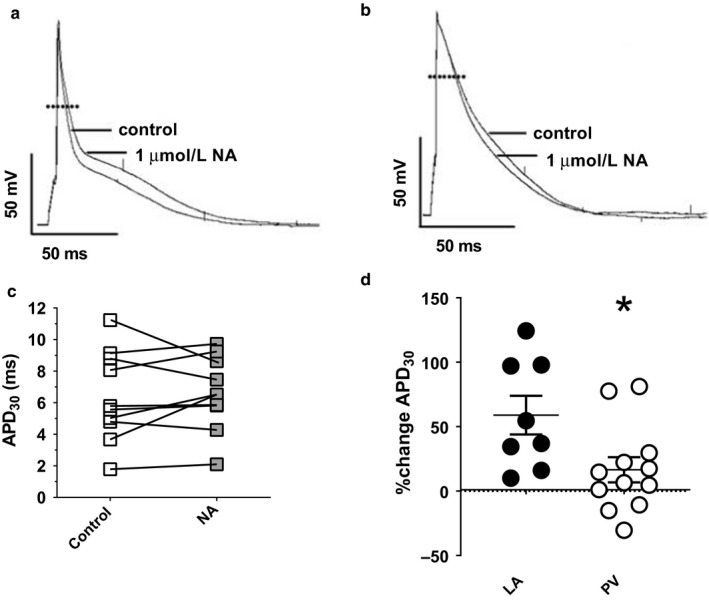
Effects of noradrenaline (NA) on action potential (AP) repolarization in PV cardiomyocytes. (a) Representative AP recording from a PV cardiomyocyte in control solution and in the presence of 1 μM NA (NA) showing prolongation of the AP. Recordings were from the same cell. (b) Representative AP recording from another PV cardiomyocyte in control solution and in the presence of 1 μM NA showing lack of AP prolongation. (c) Before‐and‐after plot of APD_30_ in PV cardiomyocytes (*n/N* = 12/10) in control solution (open squares) and after superfusion with 1 μM NA (gray squares). (d) Noradrenaline‐induced percentage changes in APD_30_ in LA and PV cardiomyocytes. LA cardiomyocytes (LA, *n/N* = 8/5, black circles), all PV cardiomyocytes (PV, *n/N* = 12/10, open circles). *p < .05; Student's *t*‐test. Bars indicate mean and standard error of the mean. The stimulus frequency for AP recordings was 1 Hz

**Figure 8:**
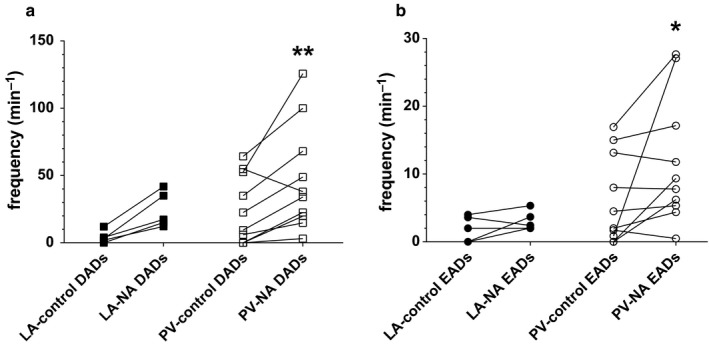
The effect of noradrenaline (1 µM) on the frequency of DADs (a, squares) and EADs (b, circles) in LA (filled symbols) and PV (open symbols) cardiomyocytes. *p < .05; **p < .01; versus corresponding control, Wilcoxon test. The data are from 5 LA cardiomyocytes from 4 rats and 10 PV cardiomyocytes from 9 rats

Noradrenaline has previously been shown to prolong APD (APD_30_) in rat isolated LA cardiomyocytes, an effect that was associated with potentiation of *I*_CaL_ and inhibition of *I*_Kss_ (Bond et al., 2014). The effects of superfusion with 1 μM NA on APD_30_ were investigated in 12 rat isolated PV cardiomyocytes (Figure 7). In 9 cells, NA caused prolongation of APD_30_ (*e.g.,* Figure 7a). However, there was a degree of heterogeneity in responses and, in three of the PV cells, APD_30_ was shorter following superfusion with NA (*e.g.,* Figure 7b). The heterogeneity in responses is evident in a before‐and‐after plot of the NA‐induced changes in APD_30_ (Figure 7c). Median APD_30_ was not changed following superfusion with NA (control median 6.9 ms, 95% confidence intervals 5.2 ms, 9.6 ms; NA median 7.0 ms, 95% confidence intervals 5.3 ms, 11.6 ms; *p* = .2036, Wilcoxon matched‐pairs signed rank test). The heterogeneity in the NA‐induced percentage‐change in APD_30_ in PV cardiomyocytes is shown in Figure 7d in comparison with the responses of 8 LA cardiomyocytes to 1 μM NA. Note that while the mean percentage‐change in APD_30_ in the LA cells was greater than 0 (*p* = .0057), as expected for a sample in which APD_30_ was increased by NA in each cell, this was not the case in the PV cardiomyocytes (*p* = .1177, one‐sampled *t*‐test). Thus, the mean NA‐induced percentage‐change in APD_30_ in PV cells was significantly less than that in LA cells (Figure 7d). Nevertheless, 9 PV cardiomyocytes showed NA‐induced percentage‐changes in APD_30_ > 0 (i.e., prolongation of APD_30_ in response to 1 μM NA), corresponding to changes in APD_30_ of similar magnitude to those shown by LA cardiomyocytes (Figure 7d). Thus, while the standard deviations of NA‐induced percentage changes in APD_30_ were similar in LA (42.4%) and PV (33.8%) cardiomyocytes, the greater heterogeneity of PV compared with LA cardiomyocytes in responses to NA was evident in the greater coefficient of variation (*CoV_s_*) in PV cells (204.1, *n/N* = 12/10) compared with LA cells (72.0, *n/N* = 8/5; *p* = .0008). In the majority of PV cardiomyocytes, the frequency of DADs and EADs was increased by 1 µM NA (Figure 8). Although the frequency of proarrhythmic events was reduced in one (for DADs, shown in Figure 8a) or two (for EADs, shown in Figure 8b) PV cardiomyocytes, the overall effect of NA in the 10 PV cardiomyocytes investigated was statistically significant (Figure 8). In contrast, while the frequency of DADs was increased in each of 5 LA cardiomyocytes, this did not reach statistical significance (Figure 8a).

**Figure 9:**
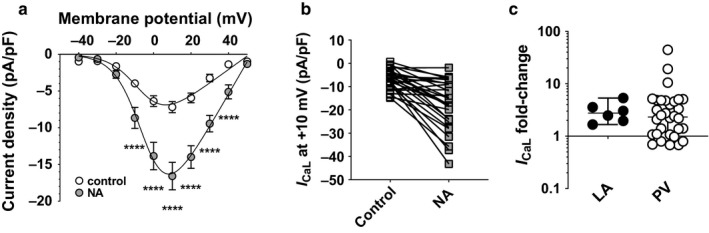
Effects of noradrenaline (NA) on *I*_CaL_ in PV cardiomyocytes. (a) Mean *I*_CaL_ density‐voltage relations of PV cardiomyocytes (*n/N* = 32/14) in control solution (open circles) and following superfusion with 1 μM NA (gray circles). Solid lines represent fits to equation 2. NA increased *G_max_* from 185 ± 21.7 pS/pF to 442 ± 55.2 pS/pF (*n/N* = 32/14, *p* < .0001). ****, *p* < .0001; two‐way RM ANOVA with Bonferroni post hoc test. Data were significantly different by membrane potential (*p* < .0001), NA (*p* < .0001) and there was a significant interaction (*p* < .0001). (b) Heterogeneity in responses to NA. Before‐and‐after plot of *I*_CaL_ at +10 mV in control and in the presence of 1 μM NA. Data as shown in panel A. Note that in 8 PV cardiomyocytes, there was a decrease in *I*_CaL_ in the presence of NA. (c) NA‐induced fold‐change in *I*_CaL_ in LA cardiomyocytes (*n/N* = 6/5, data from (Bond et al., 2014)) and in PV cardiomyocytes (*n/N* = 32/14, data as shown in panels a and b). Note the use of logarithmic scale. Bars indicate median and 95% confidence intervals

Superfusion of PV cardiomyocytes with 1 μM NA caused an ~×2.4 increase in the maximal conductance density (*G_max_*) of *I*_CaL_ (Figure 9a), very similar in magnitude to that reported previously in LA cardiomyocytes under identical recording conditions (Bond et al., 2014). However, there was considerable heterogeneity between PV cardiomyocytes in the NA‐induced changes in *I*_CaL_, as shown by the before‐and‐after plot of current density at +10 mV (Figure 9b). In 7 of 32 PV cardiomyocytes, *I*_CaL_ was *decreased* following superfusion with NA. Thus, the fold‐induced change in *I*_CaL_ density at +10 mV ranged from ×0.68 to ×44.4 (Figure 9c). In contrast, all 6 LA cardiomyocytes treated with 1 μM NA showed increases in *I*_CaL_ density, albeit over a narrower range than in PV cardiomyocytes (range ×1.67 to ×5.34). Nevertheless, there was no difference in median fold‐change in *I*_CaL_ density between LA (×2.754, 95% confidence intervals × 1.603, ×4.409) and PV cardiomyocytes (×2.225, 95% confidence intervals × 1.379, ×7.375; *p* = .4496, Mann–Whitney test), and the median fold‐changes in *I*_CaL_ were greater than 1 in both LA (*p* = .0313) and PV cardiomyocytes (*p* < .0001, Wilcoxon signed‐rank test). The greater heterogeneity of PV compared with LA cardiomyocytes in responses to NA was evident in the greater *CoV_iq_* of the NA‐induced fold‐change in *I*_CaL_ in PV cells (160.5, *n/N* = 32/14) compared with LA cells (49.0, *n/N* = 6/5; *p* = .0001).

**Figure 10:**
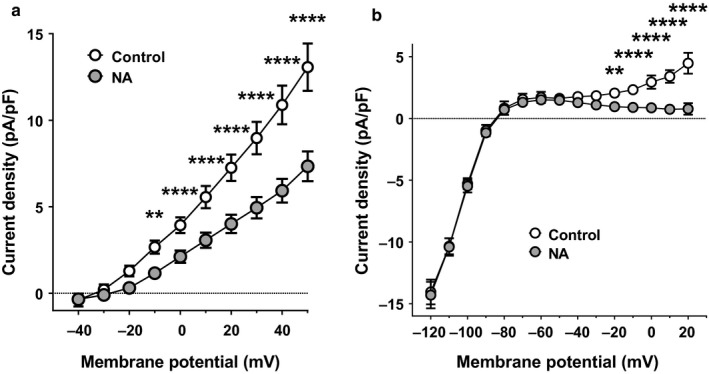
Effects of NA on K^+^ currents in PV cardiomyocytes. (a) Mean *I*_Kss_ density‐voltage relations of PV cardiomyocytes in (*n/N* = 23/11) control solution (open circles) and following superfusion with 1 μM NA (gray circles). **p < .01; ****p < .0001; two‐way RM ANOVA with Bonferroni post hoc test. Data were significantly different by membrane potential (*p* < .0001), NA (*p* < .0001) and there was a significant interaction (*p* < .0001). (b) Mean K^+^_e_‐sensitive background K^+^ current density‐voltage relations of PV cardiomyocytes in (*n/N* = 8/5) control solution (open circles) and following superfusion with 1 μM NA (gray circles). **p < .01; ****p < .0001; two‐way RM ANOVA with Bonferroni post hoc test. Data were significantly different by membrane potential (*p* < .0001), NA (*p* = .0253) and there was a significant interaction (*p* < .0001)

NA (1 μM) consistently inhibited *I*_Kss_ in PV cardiomyocytes, the current density at + 50 mV being reduced by 42 ± 4% (*n* = 23, *p* < .0001, paired Student's *t* test), as shown in rat isolated LA cardiomyocytes (Bond et al., 2014) (Figure 10a). The inhibitory effect of NA was also evident on the K^+^_e_‐sensitive *I*_Kss_ recorded at +20 mV using the ramp protocol (Figure 10b), the magnitude of which (3.7 ± 0.7 pA/pF, *n/N* = 8/5) was not significantly different from the NA‐inhibited current density at +20 mV using the square wave protocol (Figure 10a, 3.2 ± 0.5 pA/pF, *n/N* = 23/11; *p* = .646, unpaired Student's *t* test), consistent with the proposal that these effects represented the inhibition of the same population of K^+^_e_‐sensitive channels. In contrast, as shown in rat isolated LA cardiomyocytes, NA had no effect on *I*_K1_ in PV cardiomyocytes (Figure 10b) (Bond et al., 2014).

## 4 DISCUSSION

This study showed considerable heterogeneity between cardiomyocytes isolated from the pulmonary sleeves of rat hearts in the effects of NA on *I*_CaL_ and on APD_30_. The study also shows, for the first time, larger *I*_Kss_ density in PV cardiomyocytes than in left atrial cardiomyocytes. Consistent with previous studies, PV cardiomyocytes showed higher incidence of EADs, and had smaller *I*_CaL_ and *I*_K1_ densities than LA cardiomyocytes. In contrast, there were no differences in cell size, shape or capacitance, or in zero‐current potential or action potential duration between the two cell types. Noradrenaline markedly increased the frequency of DADs and EADs in PV cardiomyocytes. The heterogeneity in effects of NA on *I*_Ca_ are likely to have contributed to the heterogeneity in changes in APD_30_ and in frequency of EADs Heterogeneity in electrophysiological responses within the PVs during increased sympathetic activity may contribute to a substrate for arrhythmia.

### 4.1 Morphology of PV cardiomyocytes

PV cardiomyocytes in this study were striated rods, indistinguishable from LA cardiomyocytes under the light microscope. There were no differences between PV and LA cardiomyocytes in cell size, shape or capacitance. The rod‐shaped morphology of PV cardiomyocytes is consistent with previous reports on cells isolated from the PVs of dogs, rabbits, and rats (Chen et al., 2001; Chen, Chen, Chen, Yeh, et al., 2002; Ehrlich et al., 2003; Michelakis et al., 2001; Okamoto et al., 2012). While this study is consistent with reports from dogs and rabbits of little difference in size and shape between LA and PV cardiomyocytes (Cha et al., 2005; Chen, Chen, Chen, Yeh, et al., 2002; Ehrlich et al., 2003), previous studies in rat have shown PV cardiomyocytes to be *larger* than LA cells (Malecot, Bredeloux, Findlay, & Maupoil, 2015; Okamoto et al., 2012). In contrast, cells isolated from the rabbit PVs were reported to be *smaller* than LA cardiomyocytes (Jones et al., 2008). A marked degree of heterogeneity in cell size and morphology has been reported in some studies (Chen et al., 2001; Okamoto et al., 2012). While there was considerable heterogeneity in cell size, shape and capacitance of isolated PV cardiomyocytes in the present study, a significant degree of heterogeneity was also apparent amongst LA cardiomyocytes (Figure 2). Large, clear nodal‐like cells (large in comparison to pacemaker cells of the sinoatrial node) have been reported in the PVs of rats (Masani, 1986), similar to studies in the PVs of dogs and humans (Chou et al., 2005; Perez‐Lugones et al., 2003). Cells showing features of nodal cells, with some evidence of automaticity, have been isolated from rabbit PVs (Jones et al., 2008), although another group reported pacemaker‐like activity in rabbit isolated PV cardiomyocytes that were indistinguishable in cell size, shape or capacitance from LA cardiomyocytes (Chen, Chen, Chen, Yeh, et al., 2002). In any case, when selecting cells for recording, nodal cells would be difficult to distinguish from vascular smooth muscle cells in isolated cell preparations from the PVs (Michelakis et al., 2001). Therefore, this study focused on cardiomyocytes with a rod‐shaped, striated appearance.

### 4.2 Absence of spontaneously active cells amongst isolated PV cardiomyocytes

No phase 4 depolarizations characteristic of cells showing pacemaker activity were recorded from any of the cardiomyocytes in this study. As all the cells were paced at 1 Hz during the recording of membrane potential, we cannot exclude the possibility that the cells showed pacemaker activity at frequencies < 1 Hz. Nevertheless, it may be worth noting that none of the PV cardiomyocytes in the present study showed spontaneous activity prior to formation of the whole‐cell configuration of the patch‐clamp recording technique. Thus, the findings of the present study are consistent with previous reports that, under control conditions, there was either no spontaneous activity or that spontaneous activity was extremely slow (Cheung, 1981a, 1981b; Chou et al., 2005; Doisne et al., 2009; Ehrlich et al., 2003; Hocini et al., 2002). In contrast, cells showing automaticity have been reported from the rabbit PVs (Chen, Chen, Chen, Yeh, et al., 2002; Jones et al., 2008). The results of the present study are also in contrast to previous reports using both multicellular and isolated single‐cell preparations from rat PVs that showed spontaneous activity following superfusion with NA (Doisne et al., 2009; Maupoil et al., 2007; Okamoto et al., 2012).

### 4.3 Basal cellular electrophysiological properties

There were relatively few differences between PV and LA cardiomyocytes in the properties of the resting membrane: cell capacitances were not different and there were no differences between the two cell‐types in zero‐current potential. The zero‐current potentials in both cell‐types were depolarized in comparison to reports of resting membrane potentials in isolated PV and LA cardiomyocytes (Chen, Chen, Chen, Yeh, et al., 2002; Ehrlich et al., 2003; Okamoto et al., 2012). It is not clear what caused this, although it is unlikely to have been due to poor seal quality as recordings were only made from cells in which a seal resistance of ≥ 1 GΩ had been achieved in the cell‐attached configuration prior to whole‐cell access. One plausible explanation is the existence of a nonselective leak conductance in both cell types under the conditions of our recordings that did not contribute to the resting membrane potential in previous studies. Notably, the current‐voltage relations obtained under K^+^‐free conditions for PV and LA cardiomyocytes were almost identical, indicating that there was no significant difference between the cell types in leak conductance (Figure 5b). The density of *I*_K1_ was lower in PV than in LA cardiomyocytes, as others have reported (Cha et al., 2005; Ehrlich et al., 2003; Okamoto et al., 2012) although this was significant only at voltages negative to *E*_K_, outside the usual physiological range.

Consistent with previous studies, *I*_CaL_ density was smaller in PV than in LA cardiomyocytes (Cha et al., 2005; Ehrlich et al., 2003; Okamoto et al., 2012). The transient outward current, *I*_to_, has been suggested to be smaller in canine PV than LA cardiomyocytes (Cha et al., 2005; Ehrlich et al., 2003). However, as reported recently in rat LA cardiomyocytes, *I*_to_ made a relatively minor contribution to the whole‐cell currents or action potential repolarization of LA and PV cardiomyocytes under the conditions of this study (Bond et al., 2014). In contrast, this study showed for the first time the existence of a predominantly K^+^‐selective steady‐state outward current (*I*_Kss_) in rat PV cardiomyocytes, similar to that characterized recently in rat LA cardiomyocytes (Bond et al., 2014). Notably, *I*_Kss_ density was greater in PV than in LA cells.

The absence of marked differences between PV and LA cardiomyocytes in APD in the present study is consistent with previous reports from in vitro multicellular PV preparations from rat and dog hearts, which showed little difference in action potential repolarization at longer cycle lengths but lower APD at shorter cycle lengths reflecting a steeper AP restitution curve in PV than atrial cardiomyocytes (Cha et al., 2005; Miyauchi et al., 2005). The incidence of DADs and EADs in isolated PV cardiomyocytes is consistent with previous reports (Chen et al., 2004; Chen, Chen, Chen, Yeh, et al., 2002; Miyauchi et al., 2005). The mechanism underlying the high incidence of DADs and EADs in PV cardiomyocytes in the present study is unclear. Inward Na^+^/Ca^2+^ exchange current in response to diastolic sarcoplasmic reticulum Ca^2+^ release and T‐type Ca^2+^ current have been proposed to contribute to the high incidence of DADs in PV cardiomyocytes in previous studies (Chen et al., 2004; Hirose & Laurita, 2007; Sicouri, Glass, Belardinelli, & Antzelevitch, 2008). It has been suggested that differences between rat PV and LA cardiomyocytes in the voltage‐dependent activation and inactivation of the Na^+^ current (*I*_Na_) would lead to greater window *I*_Na_ in PV than in LA cardiomyocytes at the resting membrane potential (Malecot et al., 2015).

### 4.4 Responses to noradrenaline

NA potentiated *I*_CaL_, inhibited *I*_Kss_, and prolonged APD_30_ in PV cardiomyocytes, as has been shown in rat LA cardiomyocytes (Bond et al., 2014). To the best of our knowledge, while the effect of the β‐adrenoceptor agonist, isoproterenol, on *I*_CaL_ has been shown previously in rabbit PV cardiomyocytes (Chen, Chen, Chen, Yeh, et al., 2002), this study represents the first report of the effects of NA on *I*_CaL_ and *I*_Kss_ in PV cardiomyocytes. Responses to NA were heterogeneous, with a significant proportion of PV cells not showing APD_30_ prolongation or *I*_CaL_ increase in response to the neurotransmitter. In contrast, NA inhibited *I*_Kss_ consistently in all PV cardiomyocytes. Although inhibition of *I*_Kss_ was shown to be an important determinant of the degree of APD_30_ prolongation by NA in LA cardiomyocytes (Bond et al., 2014), taken together, these observations are consistent with the proposal that the potentiation of *I*_CaL_ plays a key role in the prolongation of APD_30_ by NA in PV cardiomyocytes. The potentiation of *I*_CaL_ by β‐adrenoceptor activation has been suggested to be key to the increase in EAD incidence in PV cardiomyocytes (Chen, Chen, Chen, Yeh, et al., 2002). Thus, the heterogeneity in *I*_CaL_ responses to NA is likely to have contributed to the heterogeneity between PV cardiomyocytes in the effects of NA on the frequency of EADs in the present study (*cf*. Figure 8 with Figure 9). In any case, it is not clear whether the increased incidence of EADs and DADs observed in the presence of NA in this study would have been evident with increased heart rate (i.e., shorter cycle lengths) as might arise in vivo with increased sympathetic activity.

The results of the present study contrast with previous reports from rat isolated single‐cell and multicellular PV preparations in which NA induced spontaneous activity (Doisne et al., 2009; Malecot et al., 2015; Maupoil et al., 2007; Okamoto et al., 2012).The reason for the difference between this study and previous studies are unclear, although it seems likely that differences in methodological approaches may have contributed to the difference in results: the majority of the studies showing spontaneous activity of the PVs in the presence of NA involved multicellular preparations and it is worth noting that NA did not induce spontaneous activity in all such preparations (Doisne et al., 2009; Maupoil et al., 2007). In contrast to the conventional ruptured‐patch whole‐cell recording technique of the present study, a perforated‐patch whole‐cell technique was used to record spontaneous activity in isolated single rat PV cardiomyocytes (Okamoto et al., 2012). Moreover, the concentration of NA used predominantly in these studies (10 μM) was higher than that used in the present study (Maupoil et al., 2007; Okamoto et al., 2012).

In addition to triggered activity, it has been suggested that heterogeneity in action potential duration within the PVs, together with changes in fiber orientation at the PV ostium, represent a substrate for reentry (Arora et al., 2003; Po et al., 2005). In this regard, heterogeneous responses to NA, if replicated in the intact PVs, could potentially contribute to the maintenance of reentry during sympathetic stimulation.

### 4.5 Limitations to the study

The relatively depolarized zero‐current potentials recorded in the present study indicated the existence of a nonselective leak conductance that is unlikely to contribute to the resting membrane in vivo. However, the injection of current to generate more negative resting membrane potentials restored membrane excitability in both PV and LA cardiomyocytes, and there was no evidence for a difference between PV and LA cells in leak conductance. This study focused on APD, *I*_Kss_, and *I*_Ca_. It is possible that there are other differences between the two cell types, outside the intended focus of this study, such as Ca^2+^ transporters and Na^+^ currents, and the possibility that these play a role in differences between PV and LA cardiomyocytes cannot be excluded. While the rat is a widely used species for the study of cardiac electrophysiology, the electrophysiological properties of rat PV cardiomyocytes, and their regulation by NA, may differ from those of human PV cardiomyocytes. Thus, this study presents novel information characterizing the electrophysiological responses to NA of PV cardiomyocytes from the rat heart.

## CONFLICT OF INTEREST

No conflicting interests, financial or otherwise, are declared by the authors.

## AUTHOR CONTRIBUTIONS

Conceptualization and research design: James, Bond. Experimentation and data acquisition: Bond, Choisy, and Bryant. Data analysis: Bond, James. Data interpretation and discussion: Bond, James, Hancox, and Choisy. Wrote or contributed to the writing of the manuscript: James, Bond, Hancox, and Choisy.
